# Supplementary material for: MetaRibo-Seq measures translation in microbiomes
Source: Nat Commun. 2020 Jun 29;11:3268. doi: 10.1038/s41467-020-17081-z (PMC7324362; doi:10.1038/s41467-020-17081-z)
Supplement: Supplementary file 10 — Supplementary Data 7 [file 41467_2020_17081_MOESM10_ESM.zip › File2/Confidence_High_Taxonomy/220622_out.krona.html]

Javascript must be enabled to view this page.

members
magnitude
magnitudeUnassigned
count
unassigned
taxon
rank

220622\_out

103

2
103
superkingdom

976
103
phylum

103
200643
class

order
171549
103

815
102
family

genus
102
816

species

SRS057717\_contig\_number\_1288
1
818

species
291644
1

SRS019787\_contig\_number\_369

821
2

SRS016495\_contig\_number\_8588SRS140492\_contig\_number\_10786
species


SRS011084\_contig\_number\_17296SRS012902\_contig\_number\_6SRS013158\_contig\_number\_33SRS013215\_contig\_number\_232SRS014459\_contig\_number\_16084SRS014613\_contig\_number\_319SRS014736\_contig\_number\_1977SRS015190\_contig\_number\_273SRS015264\_contig\_number\_2122SRS015264\_contig\_number\_11641SRS015578\_contig\_number\_3045SRS015854\_contig\_number\_980SRS015890\_contig\_number\_1838SRS015960\_contig\_number\_52SRS016095\_contig\_number\_141SRS016132\_contig\_number\_532SRS016267\_contig\_number\_182SRS017433\_contig\_number\_3889SRS017521\_contig\_number\_29308SRS017622\_contig\_number\_395SRS018313\_contig\_number\_1537SRS018541\_contig\_number\_3378SRS018656\_contig\_number\_11898SRS018817\_contig\_number\_10014SRS018888\_contig\_number\_10199SRS018984\_contig\_number\_2616SRS019267\_contig\_number\_contig-100\_1246.1246SRS019397\_contig\_number\_795SRS019601\_contig\_number\_23940SRS022524\_contig\_number\_746SRS023346\_contig\_number\_2504SRS023829\_contig\_number\_92SRS024075\_contig\_number\_1098SRS024132\_contig\_number\_11232SRS024388\_contig\_number\_302SRS024549\_contig\_number\_209SRS043701\_contig\_number\_310SRS046502\_contig\_number\_13821SRS046712\_contig\_number\_180SRS048981\_contig\_number\_574SRS050299\_contig\_number\_11549SRS051610\_contig\_number\_1766SRS053214\_contig\_number\_1524SRS053573\_contig\_number\_255SRS055966\_contig\_number\_231SRS056259\_contig\_number\_20831SRS058723\_contig\_number\_contig-100\_1408.35176SRS063040\_contig\_number\_6314SRS063127\_contig\_number\_2784SRS063190\_contig\_number\_4578SRS063985\_contig\_number\_3889SRS064276\_contig\_number\_2999SRS075021\_contig\_number\_309SRS075398\_contig\_number\_725SRS075773\_contig\_number\_19590SRS076804\_contig\_number\_9320SRS076929\_contig\_number\_contig-100\_1824.47820SRS077194\_contig\_number\_6859SRS077335\_contig\_number\_776SRS077589\_contig\_number\_1201SRS077730\_contig\_number\_397SRS078419\_contig\_number\_21787SRS098644\_contig\_number\_12033SRS098655\_contig\_number\_192SRS098881\_contig\_number\_191SRS104036\_contig\_number\_7019SRS1041039\_contig\_number\_931SRS1041145\_contig\_number\_23526SRS104311\_contig\_number\_19195SRS104485\_contig\_number\_25SRS104636\_contig\_number\_24666SRS1054928\_contig\_number\_2473SRS1055043\_contig\_number\_15997SRS1055067\_contig\_number\_1189SRS1055069\_contig\_number\_671SRS1055099\_contig\_number\_376SRS140513\_contig\_number\_8302SRS142599\_contig\_number\_8853SRS143342\_contig\_number\_3191SRS143417\_contig\_number\_1788SRS143598\_contig\_number\_1359SRS144135\_contig\_number\_8842SRS144362\_contig\_number\_1570SRS144506\_contig\_number\_20279SRS146812\_contig\_number\_49970SRS147022\_contig\_number\_62SRS147022\_contig\_number\_3720SRS147139\_contig\_number\_2437SRS147272\_contig\_number\_260SRS147377\_contig\_number\_481SRS147377\_contig\_number\_6386SRS147653\_contig\_number\_6389SRS148159\_contig\_number\_27422SRS893279\_contig\_number\_866SRS893342\_contig\_number\_25SRS971275\_contig\_number\_23108
96
817
species

species
1

SRS097920\_contig\_number\_1081
46506

species
1

SRS015065\_contig\_number\_237
28116

1
2005525
family

genus
1
375288

species
1
2292242

SRS020869\_contig\_number\_434
